# Investigating a TELEmedicine solution to improve MEDication adherence in chronic Heart Failure (TELEMED-HF): study protocol for a randomized controlled trial

**DOI:** 10.1186/1745-6215-12-227

**Published:** 2011-10-14

**Authors:** Dionne Kessing, Johan Denollet, Jos Widdershoven, Nina Kupper

**Affiliations:** 1 Center of Research on Psychology in Somatic diseases (CoRPS), Tilburg University, Tilburg, The Netherlands; 2 Department of Cardiology, TweeSteden Hospital, Tilburg/Waalwijk, The Netherlands

**Keywords:** chronic heart failure, medication adherence, telemedicine, telemonitoring, intervention trial, determinants of adherence

## Abstract

**Background:**

Frequent rehospitalisations and poorer survival chances in heart failure (HF) patients may partly be explained by poor medication adherence. There are multiple medication-related reasons for suboptimal adherence, but psychological reasons may also be important. A novel TELEmonitoring device may improve MEDication adherence in HF patients (TELEMED-HF). TELEMED-HF is a randomized, controlled clinical intervention trial designed to examine (1) the efficacy and cost-efficiency of an electronic medication adherence support system in improving and monitoring HF patients' medication adherence; (2) the effect of medication adherence on hospitalizations and health care consumption; as well as on (3) clinical characteristics, and Quality of Life (QoL); and (4) clinical, sociodemographic, and psychological determinants of medication adherence.

**Methods/Design:**

Consecutive patients with chronic, systolic HF presenting to the outpatient clinic of the TweeSteden Hospital, The Netherlands, will be approached for study participation and randomly assigned (1:1) following blocked randomization procedures to the intervention (n = 200) or usual care arm (n = 200). Patients in the intervention arm use the medication support device for six months in addition to usual care. Post-intervention, patients return to usual care only and all patients participate in four follow-up occasions over 12 months. Primary endpoints comprise objective and subjective medication adherence, healthcare consumption, number of hospitalizations, and cost-effectiveness. Secondary endpoints include disease severity, physical functioning, and QoL.

**Discussion:**

The TELEMED-HF study will provide us a comprehensive understanding of medication adherence in HF patients, and will show whether telemonitoring is effective and cost-efficient in improving adherence and preventing hospitalization in HF patients.

**Trial registration number:**

NCT01347528.

## Background

The prevalence of symptomatic heart failure (HF) is rapidly growing due to the aging of the population, successful treatment of myocardial infarction, and increased survival after an acute cardiac event [1,2]. The more frequent need for rehospitalisations and poorer survival chances in the chronic HF population are associated with major healthcare costs. For instance, almost 390 million Euros were spent on HF care in the Netherlands in 2005, and it is expected that these costs increase during the following decades [3].

Pharmacological treatment of HF typically consists of a combination of ≥4 different types of medication [4]. A major problem in HF management is poor medication adherence with rates varying from 10% to 99% [5]. Poor adherence to treatment may to a great extent explain frequent readmissions in HF patients [5], and is associated with increased morbidity and mortality [6]. A recent longitudinal study showed that medication adherence rates above 88% were associated with a reduced number of rehospitalisations and mortality rates compared to non-adherent patients [7]. In this study, objective medication adherence was measured using an electronic chip in the medication cap (*"Medication Event Monitoring"*) that records each moment the cap is removed from the medication bottle [7]. Hence, improving medication adherence may enhance cardiac prognosis, reduce the number of HF-related hospital readmissions, improve quality of life (QoL), increase survival, and reduce healthcare costs [8].

A novel tool to improve patient adherence is home telemonitoring, permitting remote patient monitoring, either by structured telephone support [9], or using telecare devices in conjunction with a telecommunication system that electronically transfers patient data [10]. Two meta-analyses have shown promising results concerning the effect of telemonitoring on improving clinical outcomes in HF patients, i.e. reducing cardiac-related hospital re-admissions and all-cause mortality by nearly one fifth, and reducing healthcare costs while improving QoL [11,12]. Only one small study tried to improve medication adherence using a specific adherence device (i.e. a medication box transferring data to an electronic record), and found that this device improved patients' compliance [10]. Most studies examining the efficacy of home telemonitoring in HF patients were either based on small samples, were time-consuming due to extensive telephone support, or did not investigate the effect of telemonitoring on longer term outcomes, or the clinical course of HF [12]. A few major randomized controlled clinical trials did use a prospective design [13,14], such as TIM-HF with a long-term follow-up of 21 months [13]. However, these trials primarily focused on improving clinical outcomes instead of improving medication adherent behavior in HF patients.

When trying to improve patients' adherence, it is important to examine potential adherence modifiers from a multidimensional perspective. Disease characteristics, regimen related factors (e.g. polypharmacy), and medication side-effects may cause people not to take their medication [15,16]. Psychosocial and socio-economic factors, such as social support, and patient-related factors, such as self-care behavior, motivation, beliefs in medication, mood, cognitive dysfunction, and personality may also affect medication adherence in HF patients [16]. Previous studies on clinical and psychosocial factors that may affect medication adherence in HF failed to find determinants of adherence [17,18]. However, these were all drug trials in which adherence was around 90%, which is not representative of the HF population in general, in which adherence rates are evidently lower.

In summary, evidence suggests that medication adherence in chronic HF patients is poor, and that it remains unclear which method(s) may be effective in improving medication adherence, ultimately improving the patient's prognosis and QoL. While some studies aimed to improve medication adherence in HF patients, the majority of the devices only recorded adherence, such as "*Medication Event Monitoring" *[7]. In the present study, the effectiveness of a novel Medication Adherence Support System (MASS) to improve medication adherence will be investigated. The MASS supplies all pills put together in one or two small bags at specified times using pharmacy-prepared medication rolls. This makes the intervention especially suitable for chronic HF patients, who are mostly elderly, care dependent, and often receive multiple different types of medication. MASS not only records adherence, but is also designed to train medication adherent behavior, and is therefore a behavior-based intervention.

### Study objectives

The primary study objectives are to (i_a_) test the efficacy and cost-efficiency of the MASS (in addition to usual care) in improving adherence as compared to usual care alone (i.e. three visits to the HF nurse); and to (i_b_) investigate the effect of medication adherence on hospitalizations and health care consumption in HF patients.

The secondary objectives are to (ii_a_) determine the effect of medication adherence on clinical characteristics (i.e., measures of disease severity, physical functioning, cognitive function), and QoL; and to (ii_b_) identify clinical, sociodemographic, and psychological determinants of subjective and objective medication adherence, while examining their role in modulating adherence and the effect of the MASS.

## Study design

The TELEMED-HF study is a Dutch, open, prospective, randomized, controlled clinical intervention trial (RCT) designed to improve medication adherence using a telemonitoring solution in a large group of HF patients. Primary and secondary outcomes will be evaluated pre- and post-intervention with a follow-up of 12 months. The trial has been registered at http://www.ClinicalTrials.gov (NCT01347528). TELEMED-HF is expected to report its first results in autumn 2013.

### Study population and eligibility criteria

Consecutive patients with a diagnosis of chronic, systolic HF presenting to the HF outpatient clinic of the TweeSteden hospital Tilburg/Waalwijk, The Netherlands, will be approached for study participation and randomized to the intervention arm (n = 200) or usual care arm (n = 200) at inclusion. Eligible patients must (a) have a diagnosis of stable HF, (b) New York Heart Association (NYHA) functional class II-III, with a decreased pump function (left ventricular ejection fraction (LVEF) < 40%), (c) titrated to the most optimal doses of ACE-inhibitor or angiotensin receptor blocker (ARB), and beta-blocker, (d) receiving stable doses of at least three HF medications for one month with no plans to add or adjust HF medications or titrate further in the immediate future, and (e) providing written informed consent.

Exclusion criteria are: (a) age < 50 years, (b) diastolic HF (intact pump function), (c) either myocardial infarction, or invasive treatment (percutaneous coronary intervention (PCI) or coronary artery bypass grafting (CABG)), or hospitalization within one month prior to inclusion, (d) participation in another clinical trial, (e) life-threatening co-morbid conditions (e.g., cancer), (f) diminished mental capacities (suspected serious cognitive decline will be confirmed by a Mini Mental State Examination (MMSE score ≤24 [19])), (g) a history of psychiatric disorders apart from affective disorders (depression and anxiety disorders), (h) significant sight and/or hearing impairment, and (i) insufficient knowledge of the Dutch language.

### Study procedures, randomization, and follow-up

The study protocol has been approved by the medical ethics committee of the TweeSteden Hospital as well as by the local regulatory authority of the TweeSteden Hospital. The trial conforms to the principles most recently outlined in the Declaration of Helsinki (2008). All patients will be informed orally and in writing about the purpose, rights, and possible benefits/risks of the study prior to participation. We have guaranteed full participation of the HF outpatient clinic of the TweeSteden hospital. Eligible patients will be invited to participate in the study during a regular visit to the HF nurse or cardiologist at the HF outpatient clinic. After 4 to 7 days patients will be contacted by telephone and asked for participation. Positively responding patients will then sign the informed consent forms and receive an appointment at the HF outpatient clinic within two weeks for their baseline visit (T0).

Patients will be randomly assigned following a blocked randomization procedure (computerized random numbers) to either the intervention arm or to usual care (1:1). Due to the restriction of a limited number of MASS devices at our disposal, the randomization procedure will be performed per week so that each week a maximum of 6 patients is assigned to either condition arms. In order to prevent selection bias, the allocation sequence will be concealed from the researcher and the HF nurse enrolling and assessing patients in sequentially numbered, opaque, and sealed envelopes. A second researcher working outside the hospital will develop and monitor the concealment system.

All patients will visit their HF nurse for their first usual care visit (T0). Corresponding envelopes based on the order of inclusion sequence will be opened only after completion of all baseline assessments during this visit to allocate the intervention. If the patient has been randomized to the intervention group, he/she will receive the MASS device and instructions, and will be fully explained how to use it. The intervention period starts within two weeks after this visit, for a period of 6 months (see below for specific details on intervention).

All patients will return to the HF outpatient clinic with a three-monthly frequency, meaning that in total three usual care visits are included in the intervention period. After the intervention period, all patients will continue to receive usual care, and will participate in four follow-up occasions at 9 (T2), 12 (T3), 15 (T4), and 18 months (T5) post-baseline that coincide with the regular three-monthly clinic appointments.

**Figure 1:**
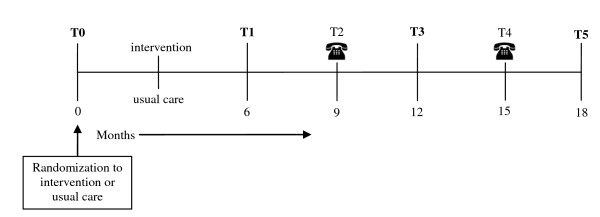
**Timeline study procedure**. The figure shows a timeline of the study procedure.

Every six months (i.e. at baseline (T0), at the end of the intervention (T1), and at 6 (T3) and 12 (T5) months thereafter), the following activities will take place in both study conditions: (i) a blood draw assessment of biochemical variables; (ii) a six-minute walk test; (iii) a NYHA functional class determination following standardized protocol; and (iv) an echocardiography to determine LVEF (%). The follow-up at 3 and 9 months will consist of a telephone call, only requesting information on healthcare consumption and adherence. The timeline of the study design is demonstrated in Figure 1.

Over the 18-month study period as well as the year before inclusion, retrospective refill rates will be requested from the patients' pharmacies. The general practitioner of the patients will be informed of the patients' participation in the study and will be asked to report the total number of patient visits to their clinic by filling out a patient contact form sent by us every 6 months.

### Study endpoints

The main study parameters include objective and subjective medication adherence, number of hospitalizations, healthcare consumption and costs (see Table 1). Objective medication adherence will be assessed using the monitor output collected in a web-based application (CarebyWeb), and the prescription refill records provided by the patients' pharmacies. A cost-benefit analysis will be assessed by means of the TiC-P questionnaire[20] and event rate (and related DBC (diagnosis treatment combination, the Dutch system to allocate costs to treatments of specific patient groups)). The TiC-P questionnaire will enable us to compare healthcare consumption in the intervention and control group.

**Table 1:** Measures & design

Construct/Measure	Methods of assessment	T0 Pre	T1 Post	T2	T3	T4	T5
*Primary outcomes*							
Adherence	Objective: Monitor output	X	X				
	Refill rate pharmacy	MR	MR		MR		MR
	Subjective: MAS, MOS-adhere	Q	Q	T	Q	T	Q
Healthcare Consumption	Number of rehospitalisations, time to first HF-related hospitalization, visits to the GP and ER due to cardiac causes, DBC codes	MR	MR	MR	MR	MR	MR
	TiC-P,	Q	Q	T	Q	T	Q
*Secondary outcomes*							
Physical function,	6-minute walk test	A	A		A		A
disease progression	NYHA, LVEF	S	S		S		S
Quality of life	KCCQ	Q	Q		Q		Q
*Predictors*							
Sociodemographic	Self-report, postal code area	Q					
Self-care behavior	EHFScBS-9	Q	Q		Q		Q
Cognitive function	MMSE	A	A		A		A
Depression	PHQ9, BDI10	Q	Q		Q		Q
Anxiety	GAD7, SAD4	Q	Q		Q		Q
Type D personality	DS14	Q	Q				

NB. Multiple instruments are used to assess anxiety and depression since there is no gold standard or consensus which instruments to use in patients with somatic disease. All psychological questionnaires are standardized and validated. Variables obtained from: S = standard measures in routine clinical care visits, A = additional assessment physical/cognitive functioning, MR = medical records, Q = questionnaire, X = intervention, T = telephone call.

The main secondary study parameters include QoL, disease severity (NYHA functional class determination following standardized protocol; LVEF using echocardiography), and exercise capacity (six-minute walk test; see also Table 1). Other secondary variables are designed to determine whether randomization was successful, to assess their potentially moderating influence on adherence, and to generate risk profiles of patients who may or may not benefit from the intervention (see Table 1). These include: (1) psychological and sociodemographic variables; i.e. Type D personality, depression, anxiety, socio-economic status, age, gender, beliefs in medication, self-care behavior, and cognitive function; and (2) clinical variables; i.e., self-reported medication side effects; time since HF diagnosis; HF etiology; previous cardiac events (MI), previous cardiac procedures (PCI, CABG), previous cardiac devices (ICD, pacemaker), number of hospitalizations for HF; blood pressure; cholesterol; triglycerides; diabetes; atrial fibrillation; comorbidities; cardiac medication, other medication (including psychotropic medication); C-reactive protein; NT-pro-BNP; parameters measured in standard clinical practice (including liver function (ACAT, ALAT, Gamma GT), hemoglobin, hematocrit, leukocytes, and kidney function (urea and creatinine).

All endpoints will be assessed with standardized and validated measures shown to have good psychometric properties, usual care laboratory tests, physical examinations, and pharmacy refill records (Table 1).

### Event adjudication

An independent, blinded, clinical endpoint committee will classify the number of all-cause and cardiac-related deaths.

### Intervention

Patients in the intervention group will be instructed to use an electronic medication adherence monitor, the MASS device, for a 6-month period and to visit the HF outpatient clinic of the TweeSteden hospital 3 times (usual care). MASS (a) dispenses all prescribed medication in the right dosage at the specified time, (b) reminds patients to take their medications through an alarm, sms or voicemail service and records adherence, and (c) sends critical data about non-adherence to the HF nurse, via a web application (CarebyWeb). The MASS device will be provided to the patient during the baseline visit, including an instruction how to use it and a manual for technical support. The patients in the intervention group will then take the monitor home and will collect a medication roll from their own pharmacy within one week. In a weekly notebook, patients will report on their perceived adherence, answer some dedicated questions on medication side-effects, satisfaction with MASS, and disease-specific and generic perceived health. After 6 months, patients will return their monitor to the researcher during their usual care visit (T1).

Recordings from the MASS device to CarebyWeb will be used as follows: (1) > 3 alerts will result into contact by the HF nurse with the patient outside regular appointments to identify reasons for not taking the medication as prescribed; and (2) if any inconsistency in medication taking behavior is being observed, the HF-nurse will use this information at the patient's next regular visit at the HF outpatient clinic to discuss reasons for non-adherence and ways to address them.

### Usual care

Patients randomized to the usual care arm will receive the care as it is normally offered consisting of three visits (0, 3, and 6 months after inclusion) to the HF outpatient clinic of the TweeSteden hospital for a consultation with the HF nurse. During this half hour visit, anamnesis will be taken, a physical examination will take place, and topics like medication adherence, and anything the patient additionally wants to discuss will be addressed. Patients will be treated for their HF in accordance with the current Dutch guideline for treatment of patients with chronic HF [4].

### Statistical analysis

This trial is designed to show whether the MASS device is effective in improving medication adherence in a chronic HF population compared to usual care only. Descriptive statistics (frequencies, means (±SD) will be calculated stratified by experimental group (intervention vs. usual care). T-tests for continuous variables and Chi-square tests for categorical variables will enable us to compare baseline values for both groups to formally test the successfulness of the randomization procedure. We will examine the effectiveness of the intervention by using the intention-to-treat principle, with the inclusion of all randomized participants in the statistical analyses regardless of whether they completed the intervention or the follow-up measurements. Missing data will be imputed using regression imputation techniques. The equality of subjective and objective adherence will be assessed by means of Intraclass correlation and Cohen's measure for agreement, the Kappa statistic, depending on the measure of adherence. Repeated measures analysis of covariance will be used to assess differences between the intervention and control group with respect to changes in adherence over the six-month intervention period, and to assess long-term effects (up to one year post-intervention) of the intervention on medication adherence, while taking potential confounders into account. To determine whether the time to hospitalization is longer for patients using the MASS device compared with patients in the control group, the log-rank test for comparison of Kaplan-Meier curves will be used. In addition, Cox proportional hazards regression modeling will be used to determine whether the usage of the MASS device predicts time to the first event (i.e. hospitalization due to HF or cardiac-related death). Multivariate regression analyses will be used to assess which psychological, clinical, demographic, and/or medication-related determinants predict adherence (with the percentage of adherence as dependent variable) in chronic HF patients and moderate the effects of the MASS device on patient adherence. The influence of potential effect modifiers will be examined by means of interaction terms.

### Statistical power analysis

The most demanding analysis in this trial with regard to statistical power and sample size is the survival analysis in which adherence is a predictor of the number of HF-related hospitalizations. A power analysis in PASS for a Cox proportional hazard regression model [21] was performed, using information from the TweeSteden hospital and previous studies. Assuming an event rate of 29% (data 2008 and 2009, TweeSteden hospital), an estimated hazard ratio of between .70 and .80 [6], taking into account an SD of 1 for our main predictor adherence, and a correlation with covariating factors of on average .30, a two-sided type I error of .05, and a minimal power of 80% to detect a HR of .70, the sample size needed for this study to be sufficiently powered is 400. In an earlier study conducted at the same outpatient clinic and within the same patient population, a response rate of 78.3% was shown with a 19.9% attrition rate [22]. Thus, in order to achieve the calculated sample size of 400 patients, we intend to include 480 patients at baseline (1:1). Based on a more conservative response rate of 70%, 685 patients need to be approached. With 400 patients visiting the outpatient clinic each year (approximately 3-5 new patients/week) inclusion of such a number within two years is feasible. This sample size is also sufficient to test the other, less power demanding, research questions.

### Subgroup analysis

We will perform subgroup analyses for the following subgroups: age (age ≤ 70 vs. > 70 years); gender (male vs. female); disease severity (NYHA class II vs. class III); dose frequency per day (1 dose vs. ≥2 doses); and cognitive function (MMSE score: highest tertile vs. all others).

## Discussion

The TELEMED-HF study is designed to test the efficacy and cost-efficiency of an innovative telemonitoring solution, the MASS device, in improving medication adherence as compared to usual care alone in a large group of HF patients. Currently, a few major clinical trials have used strategies consisting of daily telemonitoring of symptoms and health behaviors in HF patients [13,14]. However, the TELEMED-HF study is the first trial that primarily focuses on improving medication adherence by recording adherence, dispensing all prescribed medication in the right dosage at the specified times, reminding patients to take their medicines, and collecting information about non-adherence from the MASS device and responding to it. While we have chosen for a commercially available telemonitoring device, the study is designed to test its concept rather than the product itself, resembling the Tele-HF study that investigated a commercially available system on health behaviors in HF patients [23].

As the MASS device is expected to train adherent behavior, it can be considered as a behavior-based intervention. To date, most studies did not examine the effect of telemonitoring on longer term outcomes past 6 months, nor the clinical course of HF [12]. This RCT is therefore designed to determine the effect of medication adherence on the clinical state, disease progression, health care consumption, and QoL with a follow-up period of one year. We will further examine the correspondence between objectively and subjectively assessed medication adherence. Finally, our aim is to identify psychosocial and clinical determinants of medication adherence, and their role in modulating adherence and the effect of the telemonitoring device.

This intervention is innovative in comparison with other telemedicine devices. First, it supplies all pills put together in one or two small bags using pharmacy-prepared medication rolls at pharmacy-specified but patient-tailored times. Consequently, patients, partners, or informal carers do not have to prepare their medicines or a medication box weekly, reducing the chance of making possible errors. This makes the intervention especially suitable for the chronic HF population with mostly elderly patients who are often care dependent, receiving multiple different types of medication. Second, the treating physician or HF-nurse can use the information on pill taking behavior collected by the monitor to discuss reasons for non-adherence during the regular visits and if necessary, ways to address them.

The main clinical relevance of the TELEMED-HF-study is that improved adherence may lead to a better clinical condition of a chronic HF patient. This may reduce the chance for hospitalization due to heart failure deterioration, while ultimately improving the prognosis and QoL of the patient. Furthermore, we may be able to draw extensive conclusions on the effectiveness of the telemonitoring device as we will include sociodemographic, clinical, and psychological variables. Self-management programs can be tailored to patients' individual needs by delineating distinct profiles of patients who benefit from this intervention and those who may not. In the end, the results of the effectiveness of the monitor may be relevant for other research fields regarding chronic patients and/or (mildly) cognitively impaired patients following a strict medication regimen (e.g. diabetes, chronic obstructive pulmonary disease, post-stroke). Moreover, the TELEMED-HF-study is expected to fulfill the growing general need to obtain more knowledge on medication adherence in chronically ill patients, and to develop interventions from a multidimensional perspective.

From a socioeconomic perspective, the results of this trial will enable us to examine the cost-effectiveness of the intervention. Although initial costs are substantial, due to the purchase price of the MASS device and monthly rates, the subsequent reduction in health care consumption and costs may compensate these costs tremendously. Positive results may even lead to an implementation of the MASS device in clinical practice. Without the need of a special mobile telephone connection or trained personnel, the monitor could be easily provided to HF patients that are expected to benefit from this type of intervention as part of the clinical care of the HF outpatient clinic with a standardized duration depending on the cost-efficiency.

In summary, the results of the TELEMED-HF study are expected (a) to provide us a comprehensive understanding of medication adherence in HF patients, (b) to indicate whether the MASS device is effective and cost-efficient in improving medication adherence and preventing hospitalization in HF patients, and (c) to identify which subgroups of HF patients may benefit most from MASS.

## Trial Status

The TELEMED-HF trial was conceived and designed in 2010. At the time of the manuscript submission the study protocol had full ethics approval from the Medical Ethics Committee. The first participant will be randomized in October, 2011.

## List of abbreviations

HF: heart failure; MASS: Medication adherence support system; QoL: Quality of life.

## Competing interests

The authors declare that they have no competing interests.

## Authors' contributions

NK in collaboration with DK, JD, and JW designed the study. DK drafted the manuscript. NK, JW, and JD revised the manuscript critically. All authors read and approved the final manuscript to be published.
